# Vanadium Compounds as Pro-Inflammatory Agents: Effects on Cyclooxygenases

**DOI:** 10.3390/ijms160612648

**Published:** 2015-06-04

**Authors:** Jan Korbecki, Irena Baranowska-Bosiacka, Izabela Gutowska, Dariusz Chlubek

**Affiliations:** 1 Department of Biochemistry and Medical Chemistry, Pomeranian Medical University, Powstańców Wlkp. 72 Av., 70-111 Szczecin, Poland; E-Mails: jan.korbecki@onet.eu (J.K.); dchlubek@sci.pam.szczecin.pl (D.C.); 2 Department of Biochemistry and Human Nutrition, Pomeranian Medical University, Broniewskiego 24 Str., 71-460 Szczecin, Poland; E-Mail: izagut@poczta.onet.pl

**Keywords:** vanadium, tyrosine phosphatase, cell signaling, cyclooxygenase

## Abstract

This paper discusses how the activity and expression of cyclooxygenases are influenced by vanadium compounds at anticancer concentrations and recorded in inorganic vanadium poisonings. We refer mainly to the effects of vanadate (orthovanadate), vanadyl and pervanadate ions; the main focus is placed on their impact on intracellular signaling. We describe the exact mechanism of the effect of vanadium compounds on protein tyrosine phosphatases (PTP), epidermal growth factor receptor (EGFR), PLCγ, Src, mitogen-activated protein kinase (MAPK) cascades, transcription factor NF-κB, the effect on the proteolysis of COX-2 and the activity of cPLA_2_. For a better understanding of these processes, a lot of space is devoted to the transformation of vanadium compounds within the cell and the molecular influence on the direct targets of the discussed vanadium compounds.

## 1. Introduction

Vanadium compounds are known as promising drugs for lowering blood glucose in diabetes, due their insulin-mimetic properties and ability to counteract insulin resistance [1,2,3,4,5,6,7]. They are also able protect against carcinogens by increasing the activity of phase I and phase II drug-metabolizing enzymes [8,9]. In tumor cells they trigger the G_2_/M cell cycle arrest and cause apoptosis in these cells [10,11,12]. However, research into the use of vanadium compounds for treatment of tumors is still much less developed than in the treatment of diabetes [3,6,7,9].

**Figure 1:**
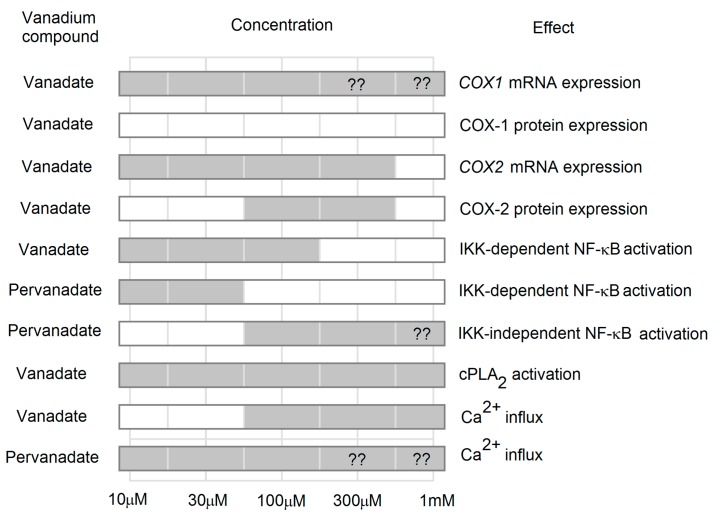
Effect of vanadium compounds on the selected processes in the expression and activity of COXs. Vanadium compounds at different concentrations cause changes (gray) in COXs protein and mRNA expression. These processes are associated with activation of NF-κB. In addition to the effects on the expression of *COX*, vanadium compounds also increase the activity of COX through the activation of cPLA_2_. This process is due to the influx of Ca^2+^, among other things. ??–unknown data

In addition to its potential medicinal properties, vanadium compounds can also cause poisonings, which constitutes another important area of research on vanadium. Its normal human blood concentrations of about 1 nM, associated with the natural presence of vanadium compounds [13], may significantly increase in the conditions of considerable anthropogenic vanadium pollution in industrialized and highly urbanized areas [14,15,16,17]. The pollution results from the combustion of oil and coal which contain large amounts of vanadium [18,19], for example, blood vanadium levels in the population of Taiwan is ca 10 nM [20] and in factory workers occupationally exposed to vanadium-containing dust may exceed 4 μM [13]. At this last concentration, vanadium compounds exhibit therapeutic properties *in vivo* in diabetic rats [4,21] and *in vitro* in human tumor cells [22]. Nevertheless, at a concentration of 7.5 μM vanadium compounds activate the Ca^2+^-dependent cytoplasmic phospholipase A_2_ (cPLA_2_), which increases the synthesis of metabolites of arachidonic acid (AA) (Figure 1) [23,24,25,26]. Prostanoids have important functions in physiology but are also an important factor in the pathology of many diseases. The main pro-inflammatory prostaglandin, prostaglandin E_2_ (PGE_2_) inhibits apoptosis and stimulates tumor cell division [27]. Therefore drugs used in tumor therapy, as well as those used in chronic diabetes management, should not increase PGE_2_ synthesis.

At much higher *in vitro* concentrations of about 100 μM vanadium compounds increase the expression of cyclooxygenase-2 (COX-2) protein [28]. Yet that high concentration of vanadium compounds *in vivo* are highly toxic, resulting in hypoglycemia, liver damage, severe acute renal failure and disrupting cellular respiration by interfering with mitochondrial function [29].

## 2. Vanadium Compounds in the Cell

**Figure 2:**
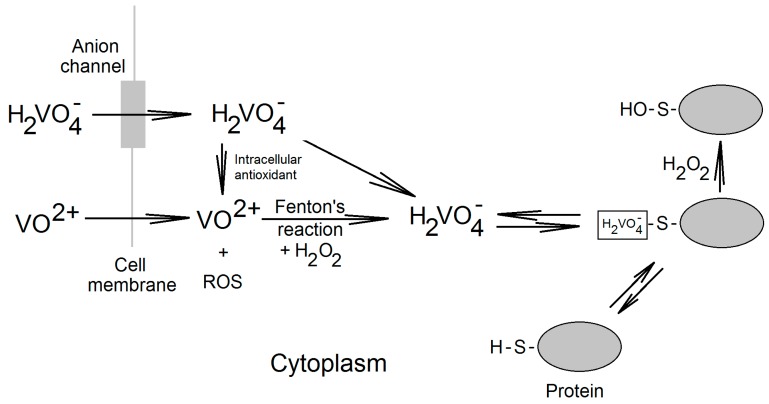
Vanadium compounds in the cell. Vanadyl and vanadate enters the cell by passive diffusion and through the anionic channels, respectively. Then, in the cytoplasm vanadyl cations may be subject to Fenton reaction in which vanadate is produced. Vanadate is reduced by intracellular antioxidants to vanadyl cations. Vanadate present in a cell does not occur in the cytoplasm, where it is bound to proteins with free cysteine residues. In the reaction with H_2_O_2_ the complexed vanadate irreversibly oxidizes cysteine residues.

In biological systems vanadium is present in +4 or +5 oxidation states and in ascidians in +3 oxidation state. Under physiological conditions, vanadium in the +4 oxidation state is present in the form of vanadyl cations (VO^2+^). In the +5 oxidation state it can be found as vanadate ions, e.g., orthovanadate (H_2_VO_4_^−^) [30]. In the bloodstream, vanadium in the +5 oxidation state enters cells through anionic channels while vanadium in the +4 oxidation state reaches cells by passive diffusion and transferrin binding vanadyl cations by endocytosis (Figure 2) [31]. In the cytoplasm, due to the reduction by the intracellular antioxidants, vanadium is present in the +4 oxidation state as vanadyl ions [32,33]. This reaction results in the formation of reactive oxygen species (ROS) which at high vanadate concentrations cause oxidative stress [33,34,35]. In the cytoplasm vanadyl cations are subsequently subject to the Fenton reaction with H_2_O_2_ to form vanadate ions and hydroxyl radical HO· [36,37]. Vanadate from this reaction enters the cell and does not appear in the cytoplasm, but instead is bound to proteins at cysteine residues [38]. In this form, vanadate and H_2_O_2_ can form pervanadate which directly oxidize thus bound cysteine residues [38,39]. This process is significant in the case of simultaneous exposure to vanadium compounds and substances that cause oxidative stress [40].

Vanadate bound to cysteine residues combines with H_2_O_2_, which causes the oxidation of the cysteine residues [38,39,40]. In many enzymes, the cysteine residues located in the active centers play essential functions in catalysis. Therefore, the oxidation of these residues by pervanadate and vanadate ions results in the inactivation of enzymes, for example protein tyrosine phosphatases (PTP), sensitive to vanadate concentrations below 1 μM [39,41,42,43]. The activity of protein tyrosine phosphatase-1B (PTP-1B) is inhibited *in vitro* by vanadate with *K_i_* = 0.38 ± 0.02 μM [39].

Nevertheless, the very mechanism of PTP inactivation depends on the type of vanadium compound. Vanadate ions, thanks to their structural similarity to phosphate anions, block the PTP catalytic center, reversibly inhibiting the PTP activity [38,39]. Another mechanism is observed for pervanadate ions, which irreversibly oxidize cysteine residues in the catalytic centers of PTPs and thus irreversibly inactivating the enzymes [39]. Vanadate may also irreversibly inactivate PTPs although to a smaller extent. Vanadate, being complexed with the catalytic cysteine residues, react with H_2_O_2_. This reaction produces pervanadate which irreversibly inactivate PTPs [39,40]. Vanadium compounds may also indirectly affect the activity of PTPs. ROS generated by the transformation reactions of vanadium compounds in the cell may inactivate PTPs by the oxidation of cysteine residues in the catalytic centers of these enzymes [44].

PTPs regulate phosphorylation of proteins in intracellular signaling pathways. Accordingly, the inhibition of these enzymes by vanadium compounds results in the activation of some signaling pathways. First of all, this means the increased phosphorylation of receptors and the mitogen-activated protein kinase (MAPK) cascades, which initiates signal transduction [45,46,47].

## 3. Effect of Vanadium Compounds on the Expression of Cyclooxygenases

### 3.1. Expression of Cyclooxygenases

Cyclooxygenase is an enzyme transforming AA into prostaglandin H_2_. Then prostaglandin is converted into prostaglandins and thromboxane by respective synthases. In humans, there are two isoforms of cyclooxygenase (COX): cyclooxygenase-1 (COX-1) and cyclooxygenase-2 (COX-2). COX-1 is a constitutive enzyme whose expression is rarely subject to change [48]. Nevertheless, in literature there are cases of increased expression of *COX1* under the influence of shear stress, phorbol esters or estrogens [49,50,51]. In contrast, COX-2 is an adaptive enzyme with a very complex regulation of expression and activity.

*COX2* expression is regulated at the stage of mRNA transcription (modification of transcript stability) and by post-translational modification of COX-2. The transcription of *COX2* mRNA involves many signaling pathways. The most important are the MAPK kinase cascades and transcription nuclear factor κB (NF-κB) [52]. The MAPK and NF-κB cascades are important for the induction of *COX2* expression by LPS, with NF-kB being an essential factor [53].

Regulation of *COX2* expression also occurs at the mRNA level. The stability of *COX2* transcription is regulated by the 3′ UTR region, which is dependent on the activation of p38 MAPK [54]. After the synthesis of COX-2, another mechanism for regulating the activity of this enzyme is possible. In contrast to COX-1, COX-2 may be phosphorylated, which results in an increased activity of this enzyme [55,56]. Nevertheless, this mechanism of COX-2 regulation is poorly understood. The final of the stages of COX-2 regulation is proteolytic degradation of this protein. In contrast to COX-1, COX-2 has a very short half-life in a cell, only several hours [48]. Degradation of COX-2 occurs through an ATP-dependent 26S proteasome [48]. The compounds of vanadium affect all stages of regulation of COX expression and activity.

### 3.2. Effect on the Expression of Cyclooxygenase-1

The vanadium-activated intracellular signaling pathways results in the expression of COX. At a concentration of 10 μM vanadate increases the expression of *COX1* mRNA, with no effect on the COX-1 protein levels [57,58]. The putative molecular mechanism of this process may involve the activation of the promoter gene *COX1*. This promoter contains a specificity protein 1 (Sp1)-binding site and the 8 intron of this gene contains activator protein-1 (AP-1)-binding site, thus increasing the transcription of *COX1* mRNA [59]. At low concentrations of about 10 μM, vanadate activates AP-1 [60]. In contrast, the transcription factor Sp1 can be activated by the MAPK cascades induced by vanadium compounds [61,62].

### 3.3. Effect on the Expression of Cyclooxygenase-2

At a concentration of about 10 μM vanadate induces the expression of *COX2* mRNA, while only the concentration of about 100 μM does it increase the expression of COX-2 protein [28,57,58]. The effect on the expression of *COX2* mRNA and protein is associated with the activation of MAPK cascades and the activation of the NF-κB [63,64,65]; the effect of each cascade on the expression of COX-2 is dependent on cell type [28,58]. In the human umbilical vein in endothelial cells (HUVEC) p38 and extracellular signal-regulated kinase (ERK) MAPK are activated [58]. It cannot be excluded that the expression of *COX2* is influenced by c-Jun N-terminal kinase (JNK) MAPK [58]. In turn, in the human lung carcinoma cell line A549 vanadium compounds enhance the expression of *COX2* by the activation of the epidermal growth factor receptor (EGFR) [28]. This receptor causes the activation of p38 MAPK cascade which is directly responsible for the expression of COX-2 in A549 cells [28].

#### 3.3.1. EGFR Signal Transduction Resulting in Increased Expression of Cyclooxygenase-2

The activity of EGFR depends on the phosphorylation of this receptor at Tyr^992^ and Tyr^1173^. In the absence of the epidermal growth factor (EGF), both these residues are dephosphorylated by PTPs; Tyr^992^ by PTP-1B and SH2 domain-containing protein tyrosine phosphatase 2 (SHP-2) [66,67], whereas the phosphorylation of Tyr^1173^ is regulated by SH2 domain-containing protein tyrosine phosphatase 1 (SHP-1) [68]. During activation of EGFR, this receptor phosphorylated, among others, at Tyr^992^ and Tyr^1173^ [47]. These phosphorylated residues bind phospholipase C-γ (PLCγ), wherein the enzyme has a higher affinity for P-Tyr^992^ [47,69]. Activated PLCγ causes the release of inositol trisphosphate (IP_3_) and diacylglycerol (DAG). This first secondary messenger causes the influx of Ca^2+^. DAG activates protein kinase C (PKC) and intracellular signal transmission to the MAPK cascades. The phosphorylation of EGFR Tyr^1173^ residue also causes a PLCγ-independent activation of MAPK cascades. The process proceeds via the activation of GTP-binding protein Ras [47,70,71].

**Figure 3:**
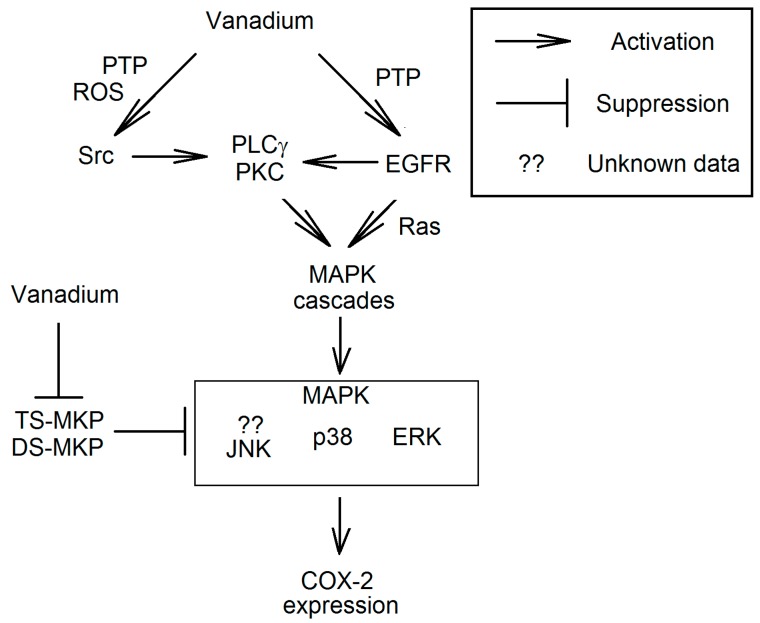
The mechanism of the activation of mitogen-activated protein kinase (MAPK) cascades in the expression of COX-2 by vanadium compounds. Vanadium compounds are the inhibitors of PTPs which directly affect the activity of Src, as well as epidermal growth factor receptor (EGFR) and MAPK. The activation of Src results in the phosphorylation of tyrosine residues on PLCγ. This process causes the binding of PLCγ to phosphorylated EGFR or to another receptor. This is followed by the transmission of the signal to PKC and consequently the activation of MAPK cascades. Vanadium compounds can activate MAPK cascades also by EGFR, independently of PLCγ.

Through the inhibition of PTP activity, vanadium compounds increase the overall phosphorylation on the EGFR tyrosine residues [47]. This causes the signal transduction to MAPK cascade (Figure 3). In human A431 squamous carcinoma cells the highest susceptibility to vanadate is shown by EGFR Tyr^992^, the phosphorylation of which results in the signal transmission by PLCγ-PKC to MAPK cascades [47]. Nevertheless, PLCγ-dependent pathway is not the only route of MAPK cascades activation. In addition, in the human airway, epithelial BEAS-2B cells vanadium compounds activate Ras, which results in the transmission of the signal to MAPK kinase cascades and NF-κB [70,71].

#### 3.3.2. Activation of MAPK Cascades Independent of EGFR

Vanadium compounds cause the expression of COX-2 by the activation of MAPK cascades via a number of pathways. In HUVEC cells, this process is EGFR-independent [58,72]. Vanadate in these cells activate non-receptor tyrosine kinases of the Src family (Src), which activate PLCγ [72]. PLCγ causes PKC signal transmission and thus the activation of the ERK MAPK cascade [72]. The ERK MAPK pathway is independent of EGFR and MAPK phosphatase (MKP) [72].

#### 3.3.3. Activation of the Src

**Figure 4:**
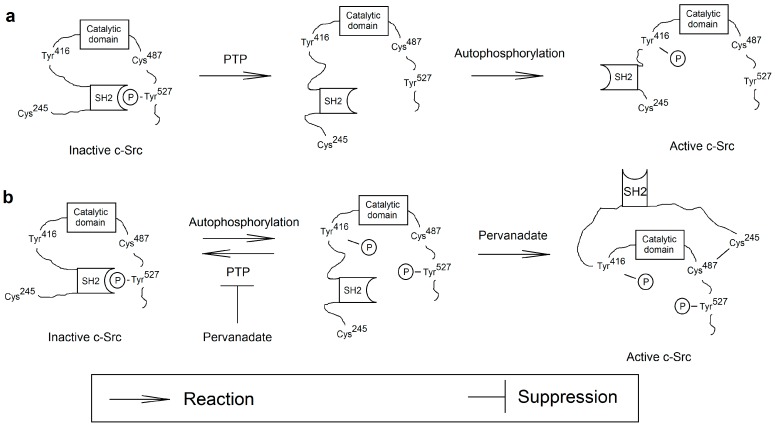
The proposed mechanism of c-Src activation of vanadium compounds. (**a**) c-Src is activated by the dephosphorylation of Tyr^527^ by PTPs. This stage is followed by the cross-autophosphorylation of c-Src at Tyr^416^ which results in the activation of these kinases; (**b**) Part of the c-Src pool in the inactive form is cross-autophosphorylated and occurs in the double phosphorylated form, at Tyr^527^ and Tyr^416^. Phosphorylation at Tyr^416^ is abolished by PTPs. Vanadium compounds as inhibitors of PTP cause the accumulation of the double-phosphorylated form. This form may be oxidized by pervanadate. In this process, a disulfide bond is created between Cys^487^ and Cys^245^, which destabilizes the inactive structure of c-Src by abolishing the binding of SH2 with phosphorylated Tyr^527^.

The activation and inhibition of Src involve PTPs which dephosphorylate tyrosine residues, significant for the regulation of these kinases. In addition, important in the regulation of Src activity are SH2 domains, which recognize phosphotyrosine residues and thus stabilize the inactive conformation of Src. The structure of Src contains two tyrosine residues that are key for the regulation of this family of kinases (Figure 4) [73,74]. In c-Src these are Tyr^527^ and Tyr^416^. After the phosphorylation of c-Src Tyr^527^, the SH2 domain of this protein binds to c-Src P-Tyr^527^, stabilizing the inactive conformation of c-Src. The activation of this kinase family consists in the dephosphorylation of P-Tyr^527^ by various PTPs [73]. After the dephosphorylation of c-Src Tyr^527^, this kinase is in the intermediate conformation, between the active and inactive conformation. Then cross-autophosphorylation occurs by another c-Src, at Tyr^416^, which induces a conformational change and the full activation of the enzyme [74,75]. c-Src inactivation involves PTP-induced dephosphorylation of residues of c-Src P-Tyr^416^ and phosphorylation of c-Src Tyr^527^ by different kinases [74]. After this modification the SH2 domain stabilizes the inactive conformation of c-Src.

Apart from phosphorylation, the regulation of Src activity may also take a different route. Src contains some oxidizable free cysteine residues in its structure [76,77]. In particular, oxidation of c-Src Cys^245^ and c-Src Cys^487^ results in the formation of a disulfide bond which in turn results in the active conformation of c-Src [76,77,78]. The mechanism of oxidation of free cysteine residues on Src to the disulfide bond is significant in the activation of this protein by high concentrations of ROS [77].

The effect of vanadium compounds on Src activity is problematic. The activation of c-Src is dependent on the dephosphorylation of P-Tyr^527^ by PTPs, enzymes inhibited by vanadium compounds [73]. Therefore, theoretically, vanadium compounds should inhibit the activation of Src. However, *in vivo*, the compounds of vanadium, in particular pervanadate, cause the activation of Src [72,79,80]. This mechanism is dependent on phosphorylation at Tyr^416^ [81]. According to a recent study, c-Src in an inactive form with P-Tyr^527^ occurs as dimers in which Tyr^416^ undergoes cross-phosphorylation [82]. Then, double-phosphorylated c-Src is dephosphorylated at Tyr^416^ by PTPs. Certainly, vanadium compounds as inhibitors of PTPs do not inhibit the activation of c-Src per se but inhibit the return of the double-phosphorylated form of c-Src to a single-phosphorylated form (at Tyr^527^), which causes the accumulation of this first c-Src form in the cell [81].

Oxidation is the second of the processes of vanadium-induced activation of Src. Vanadium compounds, in particular pervanadate at low concentrations, have oxidizing properties and thus are able to activate Src [39,72,79,80]. Pervanadate not only irreversibly inhibits PTP activity, but also contains ROS which oxidize free cysteines [39]. Therefore they can cause oxidation of Cys^245^ and Cys^487^, c-Src residues susceptible to ROS. This process gives rise to a disulfide bond which abolishes the inhibitory effect of the P-Tyr^527^ residue on the c-Src structure. This process may take place in the conformation on a double-phosphorylated c-Src, intermediate between active and inactive [82]. What causes the complete activation of Src by low concentrations of pervanadate or high concentrations of vanadate. This results in the activation of PLCγ and NF-κB and the subsequent signal transmission in intracellular communication.

#### 3.3.4. The Effect on MAPK Phosphatase

MAPKs include ERK, p38 and JNK. These are kinases activated by dual-specificity mitogen-activated protein kinase kinases (MAPKK), phosphorylating tyrosine and threonine residues on MAPK [83]. In the reverse reaction of MAPK inactivation, the phosphate residues are removed by MKP [83,84,85]. Phosphatases responsible for this reaction are divided into three groups depending on the performed reaction. The first one includes threonine phosphatases, insensitive to vanadium compounds. The remaining groups are tyrosine-specific MKPs (TS-MKP) and dual specificity MKPs (DS-MKP), the latter one catalyzing the cleavage of phosphate from phosphotyrosine and phosphothreonine residues. Similar to PTPs, DS-MKPs are sensitive to micromolar concentrations of vanadate [86,87]. Nevertheless, in the induction of *COX2* expression, the effect of vanadium compounds on the MKPs has only a marginal effect compared with other vanadium-activated pathways [28,72].

Vanadium-compounds non-specifically inactivate TS-MKPs and DS-MKPs, which results in the extended time of MAPK activation [83,84,85]. In addition, the expression of each TS-MKP and DS-MKP is tissue-specific [86,87]. Therefore, the precise route of the effect of vanadium compounds on these phosphatases depends on many factors and is difficult to specify in a simple manner. In general, however, it can be stated taht the overall effect of the compounds of vanadium on the phosphatase is the activation of MAPK or extended time of MAPK activation.

The activation of MAPK cascades may also take place in pathways other than via PTPs. The inactive form of apoptosis signal-regulating kinase 1 (ASK-1), a mitogen-activated protein kinase kinase kinase (MAPKKK) that activates JNK and p38 MAPK, binds thioredoxin which inhibits the activity of this kinase [88]. This antioxidant protein has free cysteine residues, the oxidation of which causes the activation of ASK-1. A similar mechanism occurs with the activation of the JNK MAPK in a complex with glutaredoxin [88].

#### 3.3.5. Effect on NF-κB

In addition to the activation of MAPK cascades, *COX2* expression requires the activation of NF-κB [53]. At low concentrations, vanadium compounds activate this transcription factor and it is possible that this involves the expression of *COX2*. NF-κB is a transcription factor responsible for the expression of proteins associated with inflammatory responses and cellular stress [89]. In cytoplasm, this transcription factor occurs in association with proteins inhibiting its activity as the inhibitor of NF-κB (IκB). NF-κB activation consists in the phosphorylation of serine residues of the IκBα sub-unit by IκB kinase (IKK), which results in the breakdown of the complex and the proteolytic degradation of IκBα. IKK is activated by NF-κB-inducing kinase (NIK). Then the separation of the complex NF-κB from IκB is followed by the translocation of the transcription factor to the nucleus and the transcription of NF-κB-dependent genes.

**Figure 5:**
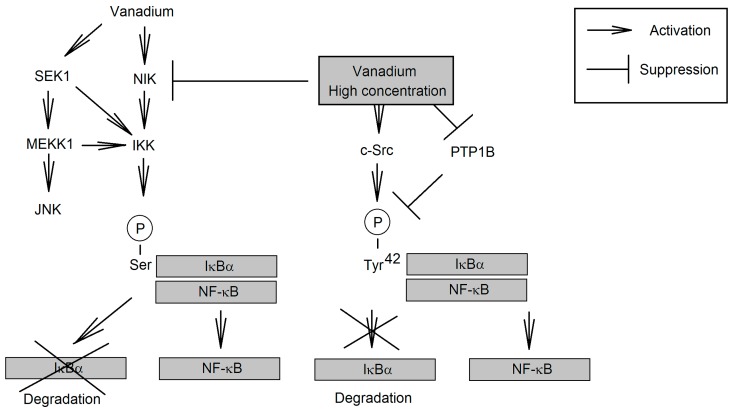
The mechanism of activation of NF-κB by vanadium compounds. Vanadium compounds at low concentrations activate NF-κB via NIK. This results in the degradation of IκBα and the release of NF-κB. The activation of IKK by vanadium compounds may also involve kinases from the JNK MAPK cascade. At high concentrations vanadium compounds have a different mechanism of NF-KB activation. They inhibit the activity of NIK and cause IκBα phosphorylation at Tyr^42^, which triggers the release of NF-κB but inhibits the proteolytic degradation of IκBα.

The described pathway leads to the activation of NF-κB by vanadium compounds at low concentrations (30 μM) [63,65,90] as well as the activation of NIK responsible for the activation of IKK (Figure 5) [64,65]. Nevertheless, the exact mechanism of NIK activation by vanadium compounds is unclear. This kinase is sensitive to changes in low concentrations of ROS and can be activated by vanadium-generated ROS [64]. It is also possible that vanadate directly activates this kinase reaction by catalyzing the H_2_O_2_-induced oxidation of cysteine residues which leads to activation of NIK [40]. Probably this process also inactivates highly ROS-sensitive serine/threonine phosphatases [64]. Another pathway of IKK activation by low concentrations of vanadium compounds are the kinases of JNK MAPK cascades, where IKK activation occurs via mitogen-activated protein kinase/ERK kinase kinase 1 (MEKK1) and SAPK/ERK kinase 1 (SEK1) [63,91].

We have described the main pathways of activation of NF-κB by vanadium compounds. Nevertheless, the transcription factor is also activated by ROS via many other pathways [92,93,94]. Thanks to the similarity in effect of vanadium compounds to ROS, vanadium may activate NF-κB in other ways, but nevertheless they may have a lot smaller significance than those described above.

At much higher concentrations (100 μM) pervanadate activates NF-κB independently of IKK through the phosphorylation of IκBα at Tyr^42^ [95,96,97,98]. This pathway is dependent on the type of cell, e.g., it takes place in T cells, due to the expression of specific kinases the activation of which is followed by the IκBα phosphorylation of proteins at tyrosine residues [80]. Probably, switching of the mechanism of NF-κB activation is associated with the inhibition of NIK activity [64]. This kinase is activated at low ROS concentrations and inactivated in severe oxidative stress by [64]. This may explain the activation of NF-κB by low concentrations of vanadium compounds (10 μM-30 μM pervanadate) and no activation or even the activation of NF-κB being inhibited in some cells by high vanadium concentrations [53,99]. In addition, the high concentrations of pervanadate inactivate PTP-1B, a phosphatase responsible for the dephosphorylation of IκBα Tyr^42^ [96]. Another mechanism responsible for the phosphorylation of this residue is pervanadate-induced activation of c-Src [80]. After the phosphorylation of IκBα at Tyr^42^, c-Src detaches from NF-κB and binds to PI3K which protects this protein against proteolytic degradation and changes in phosphorylation [95,97]. Free NF-κB is translocated to the nucleus, which is followed by the expression of genes dependent on this transcription factor.

#### 3.3.6. Effect on the Proteolysis of Cyclooxygenase-2

Proteolysis is one of the ways to change the activity of enzymes; it is also important in regulating the activity of COX-2. In the cell, this protein has a very short half-life of about a few hours [100] and its proteolysis is performed by ATP-dependent 26S proteasomes [48,100]. Thanks to the rapid degradation, the activity of COX-2 may be rapidly reduced after the removal of a proinflammatory factor.

Isolated 26S proteasomes are susceptible to the influence of vanadate at relatively low concentrations, of the order of 10 μM [101,102]. These protein complexes are ATP-dependent and therefore their activity as ATPases is inhibited by vanadate acting as phosphate analogs [30]. Nevertheless, in cellular models this effect may be observed at much higher concentrations. After getting into the cells, vanadate is reduced to vanadyl cations by intracellular antioxidants [32,33]. Therefore, vanadate concentrations inside cells are much lower than outside. This process results in the activity of 26S proteasomes being influenced by much higher vanadate concentrations in cellular models (about 100 μM or even higher) [101,102].

Synthesis of COX-2 protein is increased under the influence of a few hour long incubation of cells with vanadate [28,58]. Nevertheless, there is a lack of research in the available literature on the effect of vanadate on COX-2 protein levels after a few days of exposure. Under such conditions one could estimate the effect of vanadate on the activity of 26S proteasomes degrading COX-2 protein.

## 4. Effect on Cyclooxygenase Activity

Vanadium compounds induce the phosphorylation of tyrosine residues in a number of enzymes and thus affect their activity. This occurs via two pathways. Firstly, vanadium compounds directly inhibit PTP activity, which results in the phosphorylation of tyrosine residues in various proteins. Secondly, vanadium compounds can also cause indirect phosphorylation of proteins through the activation of specific PTP-dependent kinases. The consequence of this is the phosphorylation of enzymes, which leads to changes in their activity.

High concentrations of vanadate (at millimolar concentrations) cause phosphorylation of COX-2, which increases the activity of this enzyme [55]. The activity of another isoform, COX-1, does not depend on vanadate [55]. The exact mechanism of changes in the activity of COX-2 is not known. It is not clear which kinase is directly involved in the phosphorylation of COX-2. The most recent works show that COX-2 phosphorylation may be caused by FYN, one of Src kinases, but it is not yet known whether this kinase is associated with the action of vanadium compounds [56].

## 5. Effect on the Supply of Substrate for Cyclooxygenases

COXs catalyze the oxidation of AA to prostaglandin H_2_. The increased synthesis of this prostaglandin may be caused not only by changes in the activity of COXs, but also in the amount of available AA. An example of this is an increase in the activity of cPLA_2_ which specifically releases AA from the cell membrane [103]. The enzymes of this group are activated by the rise in cytoplasmic Ca^2+^ concentration [104]. Another pathway of cPLA_2_ activation is its phosphorylation by MAPKs.

Vanadium compounds at concentrations as low as 10 μM activate cPLA_2_, which increases the amount of released AA and the production of PGE_2_ or thromboxane A_2_ (TxA_2_) [23,24,105,106]. The activation of cPLA_2_ is complex because of the nonspecific action of vanadium compounds; it depends on the concentration, type of vanadium compound and the type of cell. The effect on MAPKs and their cascades has already been described above.

In addition, vanadium compounds activate cPLA_2_ by increased Ca^2+^ concentration [103,107,108]. Nevertheless, at low concentrations of vanadium compounds cPLA_2_ activation may depend only on MAPK. In line RAW264.7 macrophages vanadates at low concentrations (10 μM) activate cPLA_2_ only via MAPK, without changing the cytoplasmic Ca^2+^ [25]. In contrast, in FRTL-5 and NIH 3T3 fibroblasts thyroid vanadate at a concentration of 100 μM causes Ca^2+^ influx [108,109]. In addition, pervanadate at concentrations of 10 μM in different cells cause the same effect [79,103,106].

**Figure 6:**
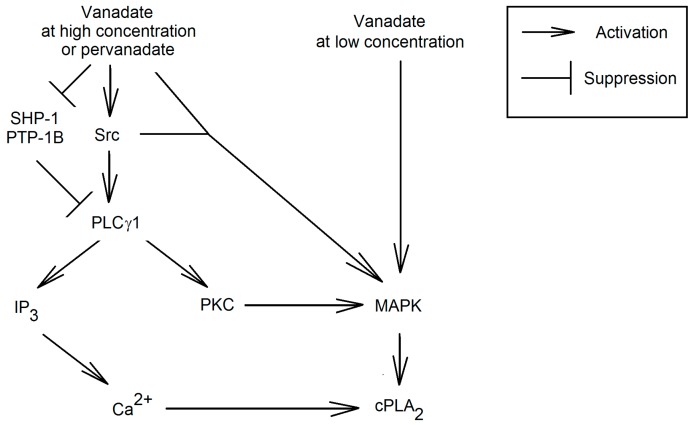
Activation of cPLA_2_ by vanadium compounds. Vanadium compounds at low concentrations activates MAPK cascades that phosphorylates cPLA_2_. At higher concentrations of vanadium compounds activate Src, which phosphorylates PLCγ1. This process causes the release into the cytoplasm of IP_3_, which causes the activation of Ca^2+^ channels and the ion influx into the cytoplasm. This PLCγ1 also activates MAPK cascades. By increasing the concentration of Ca^2+^ and the activation of the MAPK cascades is activated cPLA_2_.

The influx of Ca^2+^ induced by vanadium compounds may be due to PLCγ activation which results in the release of IP_3_ into the cytoplasm (Figure 6) [79,103,106,107,110]. Another pathway activated by PLCγ via DAG are ERK and p38 MAPK cascades, which are also involved in the activation of cPLA_2_ [104]; this route of MAPK cascade activation by vanadium compounds is tissue-specific [72,79]. IP_3_ activates ion channels that are responsible for the influx of Ca^2+^. The activation of PLCγ1 may depend on Src. Although this family of kinases may also activate PLCγ2, the influence of pervanadate via this pathway is yet to be confirmed [111].

The Src activation mechanism has already been discussed above, as well as the EGFR-dependent and independent activation of PLCγ in response to vanadium compounds. In other cells, this process can occur through other receptors. In myometrial cells pervanadate at concentrations of 10 μM activates Src which causes the phosphorylation of tyrosine residues on the platelet-derived growth factor receptor (PDGFR) and PLCγ1 [79]. Thanks to the phosphorylation of PDGFR and PLCγ1, these two enzymes form a complex because PLCγ1 has a SH2 domain that recognizes phosphotyrosine in polypeptide chains [112]. In the activation of PLCγ an important role is played by the phosphorylation of this enzyme by Src, while PDGFR serves as an anchor protein. Vanadium compounds may also activate the phospholipase by inhibiting PTPs. In astrocytes PLCγ1 is in association with SHP-1 which is inactivated by vanadate [113]. In addition, PLCγ1 activity is inhibited by PTP-1B, also sensitive to vanadium compounds [112].

The isoform of PLCγ activated by vanadium compounds depends on the type of cell. PLCγ1 is activated in HUVEC and myometrial cells [72,79,106]. This process results in the activation of cPLA_2_ and increased production of prostaglandin I_2_. However, in platelets and leukocytes vanadate activates PLCγ2, which can result in increased production TxA_2_ in platelets and thus the aggregation of these cells [103,106,110,114]. This effect in blood vessels may intensify under the influence of oxidative stress [115].

## 6. Pro-Inflammatory Properties and Therapeutic Use of Vanadium Compounds

Signaling pathways activated by vanadium compounds are well known. This makes it possible to predict the effect of vanadium compounds used in therapy and vanadium poisonings. In addition to the direct effects on inflammatory responses one should not forget about other effects caused by vanadium compounds, such as a decrease in blood glucose. In the case of vanadium compounds used in anti-diabetic therapy, this effect abolishes the pro-inflammatory effect of increased glucose concentrations [116]. Another important effect of chronic treatment with vanadium compounds is its antitumor effect [8,9].

The next step in the advancement of knowledge should be the introduction of vanadium compounds for anticancer therapy. Vanadium compounds enhance the activity of phase I and phase II drug-metabolizing enzymes and cause apoptosis and disrupt the cell division of already formed cancer cells [8]. Therefore, in *in vitro* experiments, the vanadium compounds are promising anticancer drugs, which have not yet been widely tested clinically.

Although at micromolar concentrations they increase the synthesis of PGE_2_ acting procarcinogenic, however at concentrations of 2–5 µM vanadium compounds inhibit the growth of some tumor cells [22,117]. Such vanadium concentration should be taken into consideration in anti-cancer therapy, provided the combining the long-term treatment with vanadium compounds with existing methods of anti-cancer therapy.

On the other hand, vanadium compounds also generate ROS [118] which can promote the development of diseases related to the production of free radicals and inflammatory reactions, e.g., brain neurodegenerative diseases. Therefore, the introduction of vanadium compounds to chronic diabetes management should be accompanied by the research on their effects on intracellular signaling pathways.

## Abbreviations

AA: arachidonic acid
AP-1: activator protein-1
ASK-1: apoptosis signal-regulating kinase 1
COX-1: cyclooxygenase-1
COX-2: cyclooxygenase-2
COX: cyclooxygenases
cPLA_2_: cytoplasmic phospholipase A_2_
DAG: diacylglycerol
DS.-MKP: dual specificity MKP
EGF: epidermal growth factor
EGFR: epidermal growth factor receptor
ERK: extracellular signal-regulated kinase
HUVEC: human umbilical vein in endothelial cell
IKK: IκB kinase
IP_3_: inositol trisphosphate
IκB: inhibitor of NF-κB
JNK: c-Jun N-terminal kinase
MAPK: mitogen-activated protein kinase
MAPKK: mitogen-activated protein kinase kinase
MAPKKK: mitogen-activated protein kinase kinase kinase
MEKK1: mitogen-activated protein kinase/ERK kinase kinase 1
MKP: MAPK phosphatase
NF-κB: nuclear factor κB
NIK: NF-κB-inducing kinase
PDGFR: platelet-derived growth factor receptor
PGE_2_: prostaglandin E_2_
PKC: protein kinase C
PLCγ: phospholipase C-γ
PTP: protein tyrosine phosphatases
PTP-1B: protein tyrosine phosphatase-1B
ROS: reactive oxygen species
SEK1: SAPK/ERK kinase 1
SHP-1: SH2 domain-containing protein tyrosine phosphatase 1
SHP-2: SH2 domain-containing protein tyrosine phosphatase 2
Sp1: specificity protein 1
Src: non-receptor tyrosine kinases of the Src family
TS-MKP: tyrosine-specific MKP
TxA_2_: thromboxane A_2_
